# Socioeconomic and demographic risk factors of autism spectrum disorder among children and adolescents in Bangladesh: Evidence from a cross-sectional study in 2022

**DOI:** 10.1371/journal.pone.0289220

**Published:** 2023-08-04

**Authors:** Mohammad Omar Faruk, Md. Sahidur Rahman, Md. Shohel Rana, Shohel Mahmud, Mahmuda Al-Neyma, Md. Sazzadul Karim, Nazia Alam

**Affiliations:** 1 Department of Statistics, Noakhali Science and Technology University, Noakhali, Bangladesh; 2 One Health Center for Research and Action, Chattogram, Bangladesh; 3 FETPV Technical Officer, Eastern Mediterranean Public Health Network (GHD|EMPHNET), Bangladesh Country Office, Dhaka, Bangladesh; National Research Centre, EGYPT

## Abstract

Autism spectrum disorder (ASD) is the assorted uneven conditions of the human brain that lead to developmental disabilities. This cross-sectional study aimed to identify the substantial risk factors of ASD among children in Bangladesh. The data were collected using convenience sampling through a questionnaire filled up by the trained interviewers. Mann-Whitney U and Kruskal-Wallis H tests were applied as bivariate analysis, and generalized beta regression was performed to determine the significant risk factors of autism spectrum disorder. The odds ratio (OR) along with 95% confidence interval (CI) were the measuring parameters of the risk factors of ASD. The result revealed that later birth order children have more risk of ASD (OR = 1.13, CI: 1.014–1.264, p = 0.027) compared to the children whose birth order is first. Premature birth of the child (OR: 0.87, CI: 0.76–1.00, p = 0.05) and father’s age (OR: 0.86, CI: 0.76–0.97, p = 0.020) substantially affects ASD. The maternal history of specific illness (diabetes, thyroiditis, and hypertension) during pregnancy also significantly affect ASD (OR: 1.34, CI: 1.14–1.61, p = 0.002). The results of this study would assist policymakers in taking necessary steps to reduce the incidence of this disorder by targeting the potential risk factors.

## Introduction

A neurological illness called autism spectrum disorder (ASD) impacts a child’s ability to interact, communicate, and learn [1]. An ASD patient deals with various difficulties, including sensory issues, movement impairments, learning disabilities, and mental health issues like anxiety and sadness [2]. While some people with this illness can live independently, others need care and support for the rest of their lives [3]. The prevalence of ASD has dramatically increased over the past ten years [4]. Around 52 million individuals worldwide are affected by autism to various degrees [5]. The Centers for Disease Control and Prevention (CDC) estimates that one in 44 youngsters has an ASD diagnosis, and all socioeconomic, racial, and ethnic groups can be affected by this disorder [6]. According to World Health Organization (WHO), about one out of every 100 children has ASD [7]. Recent epidemiological studies have revealed a sharp rise in the frequency of ASD, with a prevalence of four to five times higher in boys than in girls [6]. According to estimates, 1% of people in Asia, Europe, and North America have autism spectrum disorder [8, 9].

The prevalence of autism is expected to be underreported, but a serious health issue in Bangladesh [10]. Bangabandhu Sheikh Mujib Medical University (BSMMU) estimated that almost 2 in 1000 children have ASD in Bangladesh [11]. A study conducted in 2016 by the Bangladesh Ministry of Social Welfare discovered that 19% of all neurological disorders reported included autism [10]. One study recently found that the prevalence of ASD was 7.5 per 10,000 children in a rural Bangladeshi sub-district, suggesting that the prevalence of ASD in Bangladesh may be higher than previously thought [12].

ASD is linked to significant stress, worry, and loneliness in the patient’s caregivers [13]. Parental socioeconomic and cultural history plays a substantial role in developing ASD. Maternal nutrient intake and food supplements significantly impact ASD [14]. Parents with poor economic status have poor nutrition and, as a result, can have poor health for their newborn child [15]. Literature also evident that older-aged parents may have a greater risk of having a child with ASD [16]. Gestational respiratory infection, pre-term birth, and hypoxia during childbirth were the significant risk factors for ASD [17]. Gender of the child, low birth weight, urban residence, and neonatal jaundice substantially impact ASD [18]. For any prevention or control tactics to succeed, it is essential to identify the risk factors and make parents aware not to have such unexpected conditions. However, the literature revealed a scarcity of information on the risk factors of ASD in Bangladesh. Moreover, most of the studies were hospital-based investigations. Therefore, we devise a cross-sectional study throughout the central and southeastern parts of the country to investigate the potential causes of ASD among children in Bangladesh. More specifically, this study aimed to determine the neonatal, parental, and socioeconomic, risk factors of ASD.

## Methodology

### Study design

This cross-sectional study aims to assess the risk factors that significantly cause ASD among children aged 2 to 18 years in Bangladesh. Data were collected from 22 autism schools in three cities-Dhaka, Chittagong and Comilla. Dhaka is the capital city, and Chittagong is the country’s main port city. The density and number of people living in these areas are high [19], and most autism schools are also situated there (67.61%). Comilla is another city under the Chattogram division located between Dhaka and Chittagong. Therefore, this study selected these three cities. Among the selected schools, 21 were categorized as Special Educational Needs schools, while one was a mainstream school. All the schools were mixed-sex, and their student populations ranged from 15 to 150 pupils. The majority of schools (13 out of 22) were located in Dhaka, 5 schools in Chattogram, and 4 schools in Cumilla. Among these schools, 19 were non-governmental organizations, while 3 were government-run. Further details regarding the per-school breakdown can be found in S1 Appendix. The inclusion criterion for this study was autistic child aged between 2–18, and whose parents were present at the school on visiting day and gave verbal consent for the child to participate. Most of the questions were related to parents’ and children’s health conditions before and during the birth of the ASD child. Therefore, we have taken consent and interview from parents only. Children absent in school or not given consent by parents were considered exclusion criteria of this study. Parents willing to participate in the study but unable to respond to the questionnaire due to memory lapse and limited knowledge were also excluded from the survey.

### Data collection tool

After carefully revising the previous literature, the authors prepared the questionnaire for this study. The preliminary questionnaire contained 40 questions and validated the consistency through a small-scale pilot survey. Finally, a set of 36 questions was finalized for data collection. The details of the literature reviewed for constructing the questionnaire can be found in S2 Appendix. The questionnaire included information on parents’ health status during pregnancy and complications of the child during and after birth, like asphyxia, premature birth, etc. Trained interviewers collected the responses by direct interview method from the parents. The responses related to ASD were based on parents’ observations of their children. The answers for some of the independent variables were collected based on the previous health report prepared by gynaecologists and/or paediatricians. The questionnaire was designed in three major sections. The first section included neonatal information like the age of the children (number), gender (Male, Female), Birth weight (<2.5 kg, ≥ 2.5 kg), Birth order (Firstborn, Later born), Premature birth (No, Yes), Birth asphyxia (No, Yes), Breastfeeding (<6 months, 6 to 12 months, >12 months). The second section contains information on parental socio-demographic characteristics and health status. The variables included in the second section were Father’s age at the time of delivery (<40 years, ≥ 40 years), Mother’s age at the time of birth (<21 years, 21 to 35 years, >35 years), Socioeconomic status (low, middle, high), Father’s and Mother’s education (Less than secondary level, Secondary level, Higher secondary level, Graduate), Father’s occupation (Not employed, Private service, Government service, Teacher), Mother’s Occupation (Housewife, Private service, Government service, Teacher), Family types (Joint family, nuclear family), Family history of autism disorder (No, Yes), Father illness (No, Diabetes, Others (arthritis, thyroid problem, etc.)), Consanguinity (Not related, first degree related), Threatened abortion 20 weeks (No, Yes), Specific illness during pregnancy (No, Diabetes, Tyroyed, Others), Psychological stress of a mother during pregnancy (No, Yes), Maternal history of specific drug use during pregnancy (No, Yes), Poor nutrition during pregnancy (No, Yes), Vitamin D deficit (No, Yes), Mineral deficiencies (No, Yes), and Type of delivery (Vaginal devlivery, Caesarian section). Socioeconomic status was measured by key variables parents’ education, parents’ occupation, family types (joint/nuclear), present residents, and monthly family income. Monthly family income was expressed in Bangladeshi Taka (BDT) and divided into three categories- 10,000–15,000 BDT, 16,000–40,000 BDT, and >40,000 BDT. Finally, socioeconomic status was categorized into three classes- low, middle, and high, and details of the calculation of socioeconomic status would be found in S3 Appendix. In the mineral deficiency question, we only focused on Zinc deficiency as the literature has proven the role of Zinc in the neurodevelopment of infants. Vaginal delivery refers to the natural delivery of offspring either at home or in any institution without using any instrument in the presence of an expert midwife or health professionals. The information on the psychological stress of mothers during pregnancy was collected based on their self-assessment and the use of antidepressant drugs at that time. In case of threats to abortion, we asked for the history of pressure on mothers from husbands or families to abort the fetus during pregnancy which could severely affect the mother’s mental health and the development of the fetus. Poor nutrition of mother’s during pregnancy refers to mothers whose pre-pregnancy Body Mass Index score is less than 18.5, and sufficient nutrition refers to a BMI score greater than 18.5. In the third section, 10 Likert scale items were considered to assess the dependent variable (childhood autism rating score (CARSc)) and discussed in detail in the next section. The study variable considered was the score from 0 to 1 continuous scoring of ASD among Bangladeshi children.

### Assessment of childhood autism rating score (CARSc)

**Fig 1:**
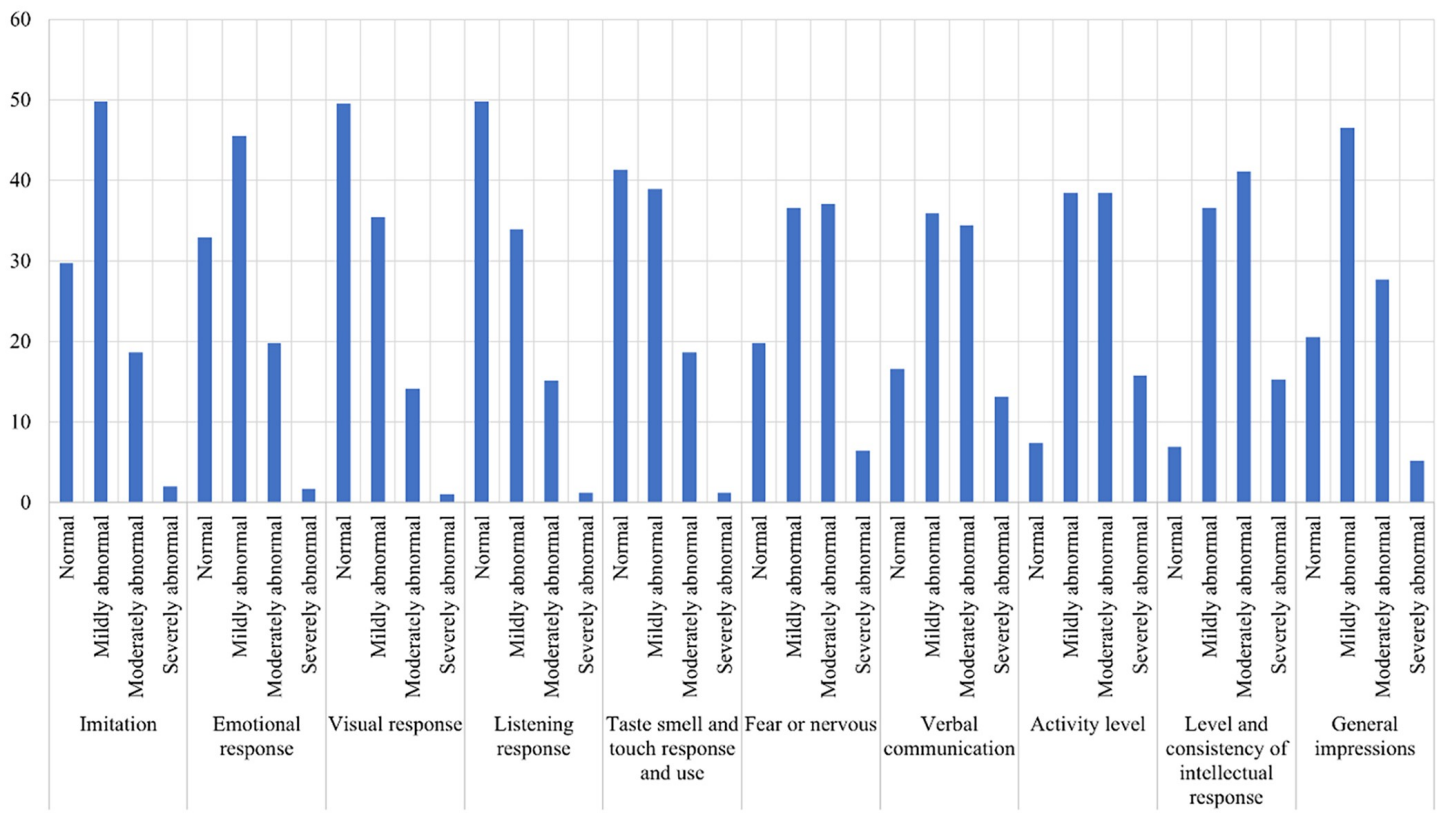
Indicators of autism spectrum disorder.

The Childhood Autism Rating Scale (CARS) is a 15-item observational rating scale to distinguish children with ASD [20] precisely. For a study, a licensed clinical psychologist or developmental pediatrician completed the CARS rating scale based on the Autism Diagnostic Observation Schedule (ADOS), parent report, and direct observation of cognitive testing [21]. A study among preschool children in Sharkia, Egypt, used the CARS to identify the risk factors of ASD [22]. The CARS tools were also successfully used in research to measure the prevalence and determinants of ASD [23–26]. The current study considered 10 items (Fig 1) out of 15 from CARS [20], eliminating and modifying some items and their coding. The details of the items removed and modified for this study can be found in S4 Appendix. Each item appeared with 4 levels coded 0, 1, 2, and 3. Where 0 indicates that the child behaves within normal limits, 1 for mildly abnormal behavior, 2 indicates moderately abnormal, and 3 for severely abnormal behavior. The items considered for the CARSc are Imitation, Emotional Response, Visual Response, Listening Response, Test, smell and touch response, Fear and nervousness, Verbal communication, Activity level, Level of intellectual response, and General Impression. By adding all 10 items, a total autism score (TAS) minimum of 0 and a maximum of 30 can be obtained. The obtained TAS was converted to a score ranging from 0 to 1 by dividing the TAS by 30 for every child. Let *V*_*ij*_ be the value of the *j*_*th*_ category of the *i*_*th*_ item responded by the parent of an autistic child. Now the total autism score (TAS) for that child can be calculated by,

$$TAS=\sum_{i}\sum_{j}V_{ij}$$ (1)

Where *i* = 1,2,3,………10 and *j* = *V* = 0,1,2,3

Now the childhood autism rating score (CARSc) can be calculated by the formula,

$$CARSc=\frac{TAS}{{TAS}_{Max}}=\frac{\sum_{i}\sum_{j}V_{ij}}{{TAS}_{Max}}$$ (2)

Here *TAS*_*Max*_ can be obtained when *j* = 3 for all *i*.

The range of the CARSc lies between 0 and 1, i.e., 0≤*CARSc*≤1. CARSc will be 0 when *j* = 0 for all *i*, and 1 when *j* = 3 for all *i*.

This score is the Childhood Autism Rating Score (CARSc). The score tending from 0 to 1 indicates that the severity of ASD increased. The details of the calculation of CARSc can be found in S4 Appendix.

#### Reliability and validity of CARSc

A scale will be helpful to a researcher if it measures some phenomenon consistently, i.e. if it is reliable. The reliability coefficient of Cronbach’s Alpha was calculated to measure the internal consistency of CARSc. The alpha value for the scale is 0.68, indicating that CARSc scale is acceptable and reliable enough [27]. The results suggest that this scale finds some unitary, essential features rather than several unrelated behaviours. Two trained psychology graduate students administrated the CARSc to assess interrater reliability. The children with autism were observed by the experimenters and completed the CARSc. Based on the ratings of 36 autistic children, the average interrater reliability obtained is 0.939. The correlation coefficient of the interrater individual items is presented in Table 1. The correlation between the total autism score and individual items score (item-total) was computed as a measure of validity. The correlation coefficients of individual items with the total score were significant (p<0.05), indicating the instrument’s validity. In summary, the measurements mentioned above support the reliability and validity of the CARSc.

**Table 1:** Interrater reliability of the individual items of CARSc.

Items	*r*_1_
Imitation	.793**
Emotional Response	.860**
Visual Response	.696**
Listening Response	.773**
Test, Smell, and Touch Response	.610**
Fear and Nervousness	.780**
Verbal Communication	.729**
Activity Level	.757**
Level of Consistency of Intellectual Response	.540**
General Impression	.853**

Note: ***r***_**1**_ is the interrater correlation of individual items, **p<0.05

### Sampling and data collection

This study used convenience sampling to select autism schools and participants for collecting data on children with ASD. Firstly, three big and busiest cities named: Dhaka, Chittagong, and Comilla in Bangladesh, were chosen. By searching on Google Maps, this study found 213 autism schools across the country, among them 144 (67.61%) schools in Dhaka, Chittagong, and Comilla. Written applications were sent for data collection to 30 schools convenient to the authors and actively running during the study period. Permission has been obtained from the authority of 22 institutions for data collection. This research used Cochran’s formula for the finite population to determine the required sample size [28]. The Cochran’s formula for sample size determination for the finite population is

$$n=\frac{n_{0}}{1+\frac{\left(n_{0}-1\right)}{N}}$$ (3)

Where N is the size of the finite population, *n*_0_ is the sample size for an infinite population, and *n*_0_ can be defined as

$$n_{0}=\frac{z^{2}pq}{e^{2}}$$ (4)

Here, *z* is the critical value of excepted confidence level, *p* is the proportion of a certain attribute presented in the population, *q* = 1−*p*, and *e* is the level of precision. Now if we consider 5% level of significance or 95% confidence interval, we have *z* = 1.96, considering the expected proportion of the study’s attribute is 50%, i.e., *p* = 0.5, *q* = 1−*p* = 0.5, *and e* = 0.05. Putting all these values in Eq (4), we can get *n*_0_ = 384.16 ≈ 384. According to Autistic Children’s Welfare Foundation, the estimated number of autistic children in Bangladesh is 3,00,000 [29]. Hence, the population size for this study is N = 3,00,000. Now, using the value of N, and *n*_0_, we have from Eq (3)

$$n=\frac{384}{1+\frac{\left(384-1\right)}{300000}}=383.51\approx 384$$ (5)

The adequate sample size for this study population is 384, and this investigation targeted 384 children for data collection. However, the study reached 450 parents, considering the possibility of incomplete response, participant withdrawal from the study, etc. Finally, we got complete responses from 404, with a response rate of 89.78%. A group of skilled and trained interviewers collected data through face-to-face interviews with the parents of the autistic child. All the data have been recorded in an Excel sheet and used for further analysis. Data collection took place between June 1, 2022, and July 31, 2022. A detailed illustration of the child with ASD considered in this study has been presented in Fig 1. As the children cannot respond to their physical, mental, socioeconomic, and demographic conditions, the data has been collected from their parents. Furthermore, the parents were informed that completing the questionnaire would take about 5–10 minutes.

### Statistical analysis

The dependent variable constructed in this study is a score variable (CARSc), which ranges from 0 to 1. The increasing value of CARSc from 0 to 1 indicates an increase in the severity of ASD. To test the normality of the outcome variable Kolmogorov-Smirnov and Shapiro-Wilk tests were applied, and the test confirmed the non-normality assumption (p<0.05) of the study variables. First, the frequency distribution of all the explanatory variables (neonatal, parental socioeconomic, and demographic features) has been constructed to understand the general information about the study. Secondly, Mann-Whitney and Kruskal-Wallis H tests have been performed as bivariate analyses. The non-parametric Mann-Whitney test was applied as the outcome variable is non-normal numeric, and the independent variables are qualitative (2 categories). Similarly, the Kruskal-Wallis H test was also applied in the same situation but when explanatory qualitative variables have 3 or more categories. The variables found significant in the bivariate analysis were further considered in the multivariate analysis. Two generalized linear regression (GLM) models: generalized linear gamma regression (GLGR), and generalized linear beta regression (GLBR) models, have been applied to assess the significant impact of different socioeconomic and demographic variables on ASD. The Akaike information criterion (AIC) has been calculated for both models to identify the best one. The AIC value for the GLBR model (AIC: -513.008) was found least compared to the GLGR model (AIC: -488.279), indicating that the generalized beta regression model is better than the gamma regression model. Hence, the results of the GLBR model have been extracted for this study and prepared the results accordingly. The software Statistical Package for Social Science (SPSS) v25 and R version 4.1.2 was used to conduct the statistical analysis.

### Ethical approval

This study was approved by Noakhali Science and Technology University Ethical Committee (NSTUEC), and the ethical approval number of this study is NSTU/SCI/EC/2022/125. Written consent was taken from the autism school authorities. As the study did not involve any medical or surgical procedure experimented on humans, verbal consent was obtained from the parents (either fathers or mothers) of ASD children. The study was conducted following the Declaration of Helsinki. Participants were ensured the confidentiality of their identity and given data. The nature of the study was completely voluntary, and no incentives were given for participation.

## Results

Fig 1 illustrates the frequency of the indicators of ASD considered in this study. The figure shows the percentage of mildly abnormal children in imitation was 49.8% (n = 201), 45.5% in emotional response (n = 184), 35.9% in verbal communication (n = 145), and 46.5% in general impression (n = 188). Again in the case of "level and consistency of intellectual response" and "fear and nervousness," the percentage of moderately abnormal children was 41.1% (n = 166), and 37.1% (n = 150), respectively.

Moreover, the number of mildly abnormal and moderately abnormal children was the same in activity level (n = 155, 38.4%). Furthermore, Fig 1 shows that almost the same number of children behave within normal limits in visual (n = 200, 49.5%) and listening responses (n = 201, 49.8%).

### Characteristics of socioeconomic and demographic features

General frequency distribution tables have been prepared to understand the characteristics of this study’s explanatory variables and are presented in Tables 2 and 3. Maximum children included in this study aged between 5 and 10 (n = 170, 42.1%) followed by the age group 11–15 years (n = 144, 35.6%) (Table 2). The table showed that 54.7% of the child (n = 221) were male, and 45.3% (n = 183) were female (Table 2). The maximum autistic child has a birth weight of over 2.5 kg (n = 312, 77.2%). Table 2 revealed that 63.4% of ASD children were firstborn (n = 256), and 64.9% (n = 262) had no birth asphyxia. Table 3 showed that 73.8% (n = 298) of fathers’ age at the time of birth of ASD-affected children was less than 40 years. The study found that 74.5% (n = 301) of mothers aged 21 to 35 and 73.5% (n = 297) of families with autistic children belong to middle socioeconomic status. In the case of Fathers’ education level, it is observed that only 3.5% (n = 14) possess less than a secondary level, 44.6% (n = 180) possess graduates, and 20.3% (n = 82) of them possess higher education.

**Table 2:** Neonatal characteristics of children with autism spectrum disorder.

Variables	Categories	Frequency	Percent
Gender	Female	183	45.3
	Male	221	54.7
Age	2–4 Years	32	7.9
	5–10 Years	170	42.1
	11–15 Years	144	35.6
	16–18 Years	58	14.4
Birth weight	< 2.5kg	92	22.8
	≥ 2.5 kg	312	77.2
Birth order	Firstborns	256	63.4
	Later born	148	36.6
Premature birth	No	254	62.9
	Yes	150	37.1
Birth asphyxia	No	262	64.9
	Yes	142	35.1
Breastfeeding	< 6 months	19	4.7
	6 to 12 months	101	25
	> 12 months	284	70.3

**Table 3:** Parental socioeconomic and demographic characteristics of autism spectrum disorder.

Variables	Categories	Frequency	Percent
Fathers’ age at the time of birth	<40 years	298	73.8
	≥40 years	106	26.2
Mothers’ age at the time of birth	<21 years	86	21.3
	21–35 years	301	74.5
	>35 years	17	4.2
Socio economic status	Low	25	6.2
	Middle	297	73.5
	Higher	82	20.3
Father’s education level	Less than secondary level	14	3.5
	Secondary level	32	7.9
	Higher secondary level	96	23.8
	Graduate	180	44.6
	Higher education	82	20.3
Mother’s education level	Less than secondary level	65	16.1
	Secondary level	68	16.8
	Higher secondary level	138	34.2
	Graduate	101	25
	Higher education	32	7.9
Father’s occupation	Not Employed	3	0.7
	Private service	140	34.7
	Government service	97	24
	Teacher	17	4.2
	Others	147	36.4
Mother’s occupation	Housewife	264	65.3
	Private service	62	15.3
	Government service	12	3
	Teacher	34	8.4
	Others	32	7.9
Family types	Nuclear family	313	77.5
	Joint family	91	22.5
Family history of autism disorder	No family history of ASD	257	63.6
	Family history of ASD	84	20.8
	Sibling history of ASD	63	15.6
Father illness	No	264	65.3
	Diabetics	45	11.1
	Others	95	23.5
Consanguinity	Not related	286	70.8
	First-degree relative	118	29.2
Threatened abortion 20 weeks	No	351	86.9
	Yes	53	13.1
Specific illness during pregnancy	No	225	55.7
	Diabetics	42	10.4
	Thyroid	24	5.9
	Others	85	21
Psychological stress of mother during pregnancy	No	115	28.5
	Yes	289	71.5
Maternal history of specific drug use during pregnancy	No	255	63.1
	Yes	149	36.9
Poor nutrition during pregnancy	No	290	71.8
	Yes	114	28.2
Vitamin D deficit	No	294	72.8
	Yes	110	27.2
Mineral deficiencies	No	286	70.8
	Yes	118	29.2
Type of delivery	Vaginal delivery	109	27
	Cesarean section	295	73

Table 3 revealed that 65.3% (n = 264) of the mothers were homemakers, and only 3% (n = 12) were government job holders. A considerable number of autistic children (n = 313, 77.5%) belong to the nuclear family, while only 22.5% (n = 91) child belongs to the joint family. The result showed that 86.9% (n = 351) of the mother was not threatened with abortion, and 63.1% (n = 255) had no maternal history of drug use during pregnancy. In addition, Table 3 revealed that 28.2% (n = 114) had poor nutrition during pregnancy, and 73% (n = 295) of the child were born by cesarean delivery.

### Bivariate analysis of the significant mean difference

Tables 4 and 5 represent the results of bivariate analysis exploring the association or significant mean rank or median difference of ASD scores among different categories of the explanatory variables. The Mann- Whitney U test in Table 4 showed no significant difference in the median score of CARSc between boys and girls (Mann-Whitney U = 19238, p > 0.05), and birth weight (Mann-Whitney U = 13378.5, p > 0.05). However, birth asphyxia was found to be significant with ASD (Mann-Whitney U = 16307, p<0.05).

**Table 4:** Mann–Whitney U test of significant mean difference of CARSc between the categories of binary explanatory variables.

			Mann-Whitney U test				
Variables	Categories	Mean	Std. Deviation	Minimum	Maximum	Test statistic	p-value
Gender	Female	0.377	0.131	0.100	0.770	19238	0.398
	Male	0.367	0.135	0.030	0.700		
Birth weight	< 2.5kg	0.390	0.153	0.130	0.730	13378.5	0.321
	≥ 2.5 kg	0.366	0.126	0.030	0.770		
Birth order	Firstborns	0.362	0.134	0.030	0.730	17045	0.092
	Later born	0.387	0.130	0.100	0.770		
Premature birth	No	0.372	0.137	0.100	0.770	18901	0.895
	Yes	0.370	0.127	0.030	0.730		
Birth asphyxia	No	0.361	0.134	0.100	0.730	16307	0.04
	Yes	0.390	0.129	0.030	0.770		
Fathers’ age at the time of birth	< 40 years	0.379	0.132	0.100	0.770	13687	0.041
	≥ 40 years	0.348	0.133	0.030	0.700		
Family types	Nuclear family	0.370	0.137	0.030	0.770	13641.5	0.539
	Joint family	0.377	0.118	0.130	0.670		
Consanguinity	Not related	0.369	0.138	0.030	0.770	16019.5	0.422
	First-degree relative	0.376	0.121	0.100	0.730		
Threatened abortion during pregnancy	No	0.364	0.132	0.030	0.770	7216	0.008
	Yes	0.420	0.133	0.130	0.730		
Psychological stress during pregnancy	No	0.334	0.118	0.130	0.630	12779	0.000
	Yes	0.386	0.136	0.030	0.770		
Maternal history of specific drug use during pregnancy	No	0.351	0.129	0.100	0.730	14412	0.000
	Yes	0.406	0.132	0.030	0.770		
Poor nutrition during pregnancy	No	0.361	0.132	0.030	0.730	14021.5	0.017
	Yes	0.397	0.133	0.130	0.770		
Vitamin D deficit	No	0.358	0.128	0.030	0.730	13075.5	0.003
	Yes	0.406	0.140	0.130	0.770		
Mineral deficiencies	No	0.357	0.126	0.030	0.700	13704.5	0.003
	Yes	0.406	0.143	0.130	0.770		
Type of delivery	Vaginal delivery	0.375	0.140	0.100	0.730	15990	0.933
	Cesarean section	0.370	0.130	0.030	0.770		

**Table 5:** Kruskal–Wallis H test of significant mean difference of CARSc among the multiple categories (more than two) explanatory variables.

		Kruskal-Wallis H test					
Variables	Categories	Mean	Std. Deviation	Minimum	Maximum	Test statistic	p-value
Breastfeeding	< 6 months	0.342	0.178	0.030	0.700		
	6 to 12 months	0.370	0.128	0.100	0.730	1.172	0.557
	> 12 months	0.374	0.132	0.100	0.770		
Mothers age at the time of birth	< 21 years	0.367	0.142	0.130	0.730		
	21–35 years	0.372	0.129	0.030	0.770	0.777	0.678
	>35 years	0.377	0.158	0.170	0.700		
Socio economic status	Low	0.355	0.167	0.170	0.730		
	Middle	0.365	0.130	0.030	0.770	5.32	0.07
	Higher	0.399	0.131	0.100	0.700		
Fathers education	Less than secondary level	0.371	0.200	0.100	0.730		
	Secondary level	0.395	0.137	0.130	0.630	4.637	0.327
	Higher secondary level	0.373	0.142	0.130	0.700		
	Graduate	0.377	0.114	0.100	0.730		
	Higher education	0.347	0.145	0.030	0.770		
Mothers education	Less than secondary level	0.370	0.171	0.100	0.730		
	Secondary level	0.373	0.116	0.130	0.630	4.355	0.36
	Higher secondary level	0.380	0.109	0.100	0.670		
	Graduate	0.368	0.128	0.100	0.730		
	Higher education	0.339	0.184	0.030	0.770		
Fathers occupation	Not Employed	0.422	0.201	0.230	0.630		
	Private service	0.357	0.146	0.030	0.770	7.128	0.129
	Government service	0.358	0.117	0.100	0.730		
	Teacher	0.349	0.087	0.200	0.500		
	Others	0.394	0.131	0.100	0.730		
Mothers occupation	Housewife	0.367	0.143	0.030	0.770		
	Private service	0.381	0.112	0.130	0.670	3.092	0.543
	Government service	0.347	0.111	0.170	0.530		
	Teacher	0.364	0.119	0.100	0.600		
	Others	0.399	0.110	0.200	0.670		
Family history of autism disorder	No family history of ASD	0.372	0.138	0.100	0.730		
	Family history of ASD	0.362	0.123	0.030	0.670	0.205	0.903
	Sibling history of ASD	0.382	0.126	0.100	0.770		
Father illness	No	0.363	0.137	0.030	0.770		
	Diabetics	0.371	0.129	0.130	0.700	3.810	0.149
	Others-thyroiditis	0.393	0.122	0.030	0.770		
Specific illness during pregnancy	No	0.347	0.125	0.100	0.700		
	Diabetics	0.365	0.119	0.170	0.630	26.145	0.000
	Thyroid	0.457	0.154	0.170	0.770		
	Others (Hypertension)	0.388	0.132	0.030	0.730		

From Table 4, it is also observed that psychological stress (Mann-Whitney U = 12779, p<0.05) and a history of specific drug use during pregnancy (Mann-Whitney U = 14412, p<0.05) were significant with ASD. The father’s age at birth of the child was found to be significant with ASD (Mann-Whitney U = 13687, p<0.05), and the highest mean ASD score was observed in the children whose father’s age was less than 40 at the time of birth (0.379±0.132). A significant difference is found between the autistic children whose mothers are threatened (and not threatened) to abortion after 20 weeks of pregnancy (Mann-Whitney U = 7216, p<0.05). Moreover, the table showed that poor nutrition during pregnancy (Mann-Whitney U = 14021.5, p<0.05), vitamin D deficit (Mann-Whitney U = 13076.5, p<0.05), and mineral deficiencies (Mann-Whitney U = 13704.5, p<0.05) were also significant with the score of ASD. Mothers with a history of drug use during pregnancy have a greater mean CARSc score (0.406±0.132).

In addition to the Mann-Whitney U test, the Kruskal-Wallis H test for testing the significant mean rank difference of CARSc has been calculated and presented in Table 5. Moreover, the results showed a significant mean rank difference in ASD scores was found in different illnesses (Kruskal-Wallis H = 26.145, p<0.05) during pregnancy. Other variables like parents’ education, occupation, mother’s age, and breastfeeding were not significant with CARSc.

### Impact of neonatal, parental socioeconomic, and demographic factors on ASD

Table 6 represents the impact of socioeconomic and demographic factors on ASD. Birth order significantly impacts ASD (OR: 1.142, p<0.05). Babies born in the second and later order are 1.142 times more at risk of having ASD compared to the baby born in 1^st^ order. Premature birth also impacted ASD (OR: 0.872, p = 0.05) significantly. This study found that babies with premature birth have less risk of ASD compared to those children who were not at premature birth.

**Table 6:** Impact of neonatal, parental socioeconomic, and demographic factors on autism spectrum disorder (Generalized Linear Beta Regression Model).

Variables	Estimates	St. Error	Z-value	Odds Ratio	95% CI of Odds		p-value
					Lower	Upper	
Intercept	-0.750	0.140	-5.37	0.472	0.359	0.621	0.000
Birth Weight (≥2.5 kg)	-0.109	0.066	-1.66	0.896	0.788	1.020	0.096
Birth Order (Later born)	0.124	0.056	2.213	1.132	1.014	1.264	0.027
Premature Birth (Yes)	-0.136	0.070	-1.95	0.873	0.761	1.001	0.051
Birth Asphyxia (Yes)	0.040	0.073	0.546	1.040	0.902	1.200	0.585
Father’s age at the time of Birth (≥40 years)	-0.149	0.064	-2.32	0.862	0.760	0.977	0.020
Socioeconomic status (Middle)	0.083	0.121	0.681	1.086	0.856	1.378	0.496
Socioeconomic status (Higher)	0.252	0.133	1.900	1.286	0.992	1.668	0.057
Threatened to abortion (Yes)	0.117	0.086	1.370	1.124	0.951	1.329	0.171
Specific illness during pregnancy (Diabetics)	0.009	0.093	0.101	1.010	0.841	1.212	0.919
Specific illness during pregnancy (Thyroiditis)	0.117	0.078	1.502	1.124	0.965	1.310	0.133
Specific illness during pregnancy (Others- Hypertension,)	0.292	0.094	3.109	1.339	1.114	1.609	0.002
Psychological stress during pregnancy (Yes)	0.086	0.066	1.306	1.090	0.958	1.240	0.192
Maternal history of specific drug use during pregnancy (Yes)	0.093	0.068	1.365	1.098	0.960	1.256	0.172
Poor nutrition during pregnancy (Yes)	-0.079	0.117	-0.672	0.924	0.735	1.163	0.502
Vitamin D deficit (Yes)	0.157	0.130	1.204	1.170	0.906	1.509	0.228
Mineral deficiency (Yes)	0.078	0.093	0.834	1.081	0.900	1.298	0.404

The odds ratio and p-value in Table 6 revealed that fathers aged greater than 40 years had less risk of having a baby with ASD. Moreover, the odds ratio of socioeconomic status (higher class) is 1.323, and the p-value is 0.057, indicating socioeconomic status has no significant impact on ASD. It is observed from the results that the wealthiest respondents have an increased risk of having a child with ASD. The people who are of higher class are 1.323 times more prone to have autistic children than those with low socioeconomic status. Furthermore, maternal history of specific illness during pregnancy significantly impacts ASD (OR: 1.033, p< 0.05). Mothers with a history of specific diseases during pregnancy are 1.339 times more at risk of having an autistic child than their mothers who do not have such illness during pregnancy.

## Discussion

The study focused on the effect of socioeconomic, demographic, nutritional, parental, and birth-related factors on the occurrence of autism spectrum disorder among children in Bangladesh. The results revealed that the birth order significantly impacts ASD, and later-born children had a significantly higher risk of having ASD than the first baby. A previous study identifying the effect of birth order and birth interval on ASD found a substantial correlation between ASD and birth order [30]. Another study conducted in California on sibling birth showed that ASD prevalence in second-born children was higher than in firstborn children, especially when they were born within 24 months [31]. The literature revealed that birth order was significantly associated with ASD phenotype, and with the increase in birth order, mental retardation, intelligence score, and adaptive functioning decreased [32, 33]. Another research conducted in West Bengal, India, found that children with high birth order have a severe phenotype of autism, while the child born in first order has less phenotype of ASD [34].

In addition, the study showed the association of paternal age with the risk for ASD. A study in New York City conducted by Quinlan and colleagues (2015) [35] found a significant association between paternal age and ASD, and the risk of ASD increased with the increase in paternal and maternal age [35, 36]. Research on the population of Thailand also found a role of advanced paternal age (age >35 years) and unemployed mothers in ASD [37]. In contrast, an investigation on 132,271 individuals conducted in Israel showed that advancing paternal age had a significant monotonic association with the risk of ASD. Idring S. et al. surveyed 417 303 Swedish children born between 1984–2003 and elucidated that older-aged fathers have a greater risk of having a child with ASD [38]. In line with the results of this study, another study found a significant association between paternal age and a greater risk of ASD in their offspring [39].

This investigation observed that parents’ socioeconomic status had no substantial impact on ASD. Similarly, a European study found no statistically significant relationship between the family’s income status and the formation of ASD in children [31]. A study examining the association between maternal education and ASD in Finland elucidated that socioeconomic status was not substantially associated with children’s autism disorder [40]. The finding of this study is consistent with the result of an investigation that the parental wealth index has no significant association with children’s ASD [41]. In contrast, a significant effect of the family’s socioeconomic level on ASD was observed in previous literature from the United States of America (USA) [42]. Another study also revealed that with the variation in socioeconomic status, the level of ASD also varied significantly [43].

The bivariate analysis showed that maternal drug exposure (Antidepressant drugs and Opioids) during pregnancy substantially impacts ASD. The mean score of ASD was observed to be high among mothers who used the specific drug during pregnancy. A population-based cohort study during 2001–11 in Stockholm County, Sweden, reported the potential impact of the use of different medications on the outcomes of ASD, particularly antidepressant drugs [44] and serotonin reuptake inhibitors [45]. Moreover, Wood AG, et., al, (2015) demonstrated that using folic acid supplements and marijuana in the first trimester of pregnancy increased childhood autism [46]. In contrast to the result of this study, an investigation observed contradictory results and showed no association between maternal drug exposure and childhood ASD [47].

The study found the psychological stress of mothers during pregnancy is a risk factor for ASD in children. The impact of psychological stress on a mother during pregnancy and the child’s early stage is also stated in a study conducted by TB Franklin and colleagues [48]. A review of environmental risk factors on ASD by L Liu, et., at, (2016) revealed that the period of 21–32 weeks of gestation is critical for the brain development of infants, and therefore stresses in this period could increase the risk of ASD [49]. On the other hand, a study in Thailand found a relationship between psychological disorders from the paternal side of the child’s family and ASD [37]. The Mann-Whitney U test revealed that poor nutrition and vitamin D deficit during pregnancy were significant for the development of ASD in children. In Perth, Western Australia, 743 Caucasian women were observed for vitamin concentration showed similar results and proved that vitamin D deficiency in the mother during pregnancy leads to the development of ASD in the newborn baby [50]. Vitamin D deficits cause speech and language problems and attention disorders in the offspring [51] and increase the risk of ASD [52].

Moreover, this study revealed that maternal history of specific illnesses during pregnancy significantly impacts ASD. Hypertension and other related diseases during pregnancy, and delivery play an important role in ASD among their offspring [53]. A survey of 15,462 children in the United Kingdom showed that reported maternal infections are significantly associated with ASD. Another investigation found that hypertension during pregnancy was significantly associated with offspring ASD [54]. An analysis of the impact of the mother eating disorder on ASD in Sweden determined that the maternal eating disorder is significantly associated with ASD in their offspring [55].

The study did not include a control group of children to measure the contribution of each risk factor for the development of ASD, which was a limitation of the study. In addition, the study had questions about the health status of parents and children during pregnancy and birth of the child, and the response could arise recall bias. Moreover, lack of randomization during the selection of schools and children may create recruitment bias and confounding. The scarcity of previous literature in the study area also limits the logical justification of the study findings. On the other hand, the study’s strength was the significant amount of data collected from ASD children from different cities in Bangladesh. Moreover, the use of CARSc to identify the risk factors of ASD is novel in Bangladesh. However, the study recommends that future investigations should focus on case-control analysis and the effect of environmental variables on ASD.

## Conclusion

Autism spectrum disorder is neglected, with insufficient epidemiological evidence for the higher incidence in Bangladesh. This cross-sectional study investigates the potential causes of ASD among children in Bangladesh. Results revealed that the birth order of the children significantly impacted ASD, and children with second and later birth order possess a higher risk of having ASD compared to the first birth. In addition, the Father’s age at birth of the child and the maternal history of specific illnesses during pregnancy posed a greater risk of having a child with ASD. The study provides baseline information for the scientific community to conduct further comprehensive analytical studies in a region with limited resources.

## Supporting information

S1 Appendix Per school breakdown or details of the selected autism schools. (DOCX)

S2 Appendix The literature reviewed for the construction of the questionnaire. (DOCX)

S3 Appendix Assessment of socioeconomic status. (DOCX)

S4 Appendix Items modified and added in the CARSc from CARS, and calculation of CARSc. (DOCX)
