# Supplementary material for: Socioeconomic and demographic risk factors of autism spectrum disorder among children and adolescents in Bangladesh: Evidence from a cross-sectional study in 2022
Source: PLoS One. 2023 Aug 4;18(8):e0289220. doi: 10.1371/journal.pone.0289220 (PMC10403138; doi:10.1371/journal.pone.0289220)
Supplement: S1 Appendix — (DOCX) [file pone.0289220.s001.docx]

**S1 Appendix:** Per school breakdown or details of the selected autism schools.

**Characteristics of the Selected Schools**

Before the collection of data, the consent of school authorities and parents was obtained. The study comprised a total of 404 children with autism spectrum disorder (ASD) from 22 schools that provided educational support specifically for children with ASD. Among the selected schools, 21 were categorized as Special Educational Needs schools, while one was a mainstream school. All the schools were mixed-sex, and their student populations ranged from 15 to 150 pupils. The majority of schools (13 out of 22) were located in Dhaka, 5 schools in Chattogram and 4 schools in Cumilla. Among these schools, 19 were non-governmental organizations, while 3 were government-run. Summary characteristics of the schools were shown in Table.

**Table A** Characteristics of Schools

| **Characteristics** | | **Frequency (n)** |
| --- | --- | --- |
| Location | Dhaka | 13 |
|  | Chattogram | 5 |
|  | Cumilla | 4 |
| Administration/Organization | Non-government organization | 19 |
|  | Government organization | 3 |
| Types of school | Special Educational Needs (SEN) | 21 |
|  | Mainstream school | 1 |

**Table B** Details information regarding the selected schools

| SL | Sch-  ool  Code | School Name | Location | Established year | Types of school | Administration/Organization | Number of respondents | Number of students enrolled | Requested parents for consent | Number of parents gave consent |
| --- | --- | --- | --- | --- | --- | --- | --- | --- | --- | --- |
| 1 | 1 | Autism Welfare Foundation (AWF) | Dhaka | 2004 | Special Educational Needs (SEN) | Non-government organization | 25 | 60 | 30 | 25 |
| 2 | 3 | Autistic Children's Welfare Foundation (ACWF) | Chattogram | 2007 | Special Educational Needs (SEN) | Non-government organization | 16 | 40 | 18 | 16 |
| 3 | 4 | Beautiful Mind | Dhaka | 2004 | Special Educational Needs (SEN) | Non-government organization | 20 | 55 | 22 | 20 |
| 4 | 5 | Odessy School for Autistic | Cumilla |  | Special Educational Needs (SEN) | Government organization | 10 | 25 | 11 | 10 |
| 5 | 6 | Matrisneha Autism Children Academy | Chattogram |  | Special Educational Needs (SEN) | Non-government organization | 6 | 20 | 10 | 6 |
| 6 | 7 | Nishpap Autism Foundation | Chattogram | 2010 | Special Educational Needs (SEN) | Non-government organization | 12 | 30 | 15 | 12 |
| 7 | 8 | PFDA-Vocational Training Center | Dhaka | 2014 | Special Educational Needs (SEN) | Non-government organization | 9 | 20 | 13 | 9 |
| 8 | 10 | School for Gifted Children | Dhaka |  | Special Educational Needs (SEN) | Non-government organization | 23 | 50 | 25 | 23 |
| 9 | 11 | Proyash School | Cumilla | 2006 | Special Educational Needs (SEN) | Non-government organization | 6 | 28 | 10 | 6 |
| 10 | 12 | Seher Autism Center | Chattogram | 2010 | Special Educational Needs (SEN) | Non-government organization | 7 | 35 | 9 | 7 |
| 11 | 14 | Society for the Welfare of Autistic Children (SWAC) | Dhaka |  | Special Educational Needs (SEN) | Non-government organization | 32 | 80 | 34 | 32 |
| 12 | 15 | Society for the Welfare of the Intellectually Disabled (SWID) | Dhaka | 1978 | Special Educational Needs (SEN) | Non-government organization | 26 | 65 | 27 | 26 |
| 13 | 16 | William & Mary Taylor Inclusive School | Dhaka | 1996 | Mainstream school | Non-government organization | 30 | 150 | 32 | 30 |
| 14 | 17 | ASSC Special School | Dhaka | 2010 | Special Educational Needs (SEN) | Non-government organization | 26 | 50 | 29 | 26 |
| 15 | 19 | Special Care Foundation (SCF) | Dhaka | 2015 | Special Educational Needs (SEN) | Non-government organization | 27 | 60 | 27 | 27 |
| 16 | 20 | Comilla Apollo Information Centre | Cumilla |  | Special Educational Needs (SEN) | Non-government organization | 8 | 30 | 10 | 8 |
| 17 | 21 | Smiling Children Special School | Dhaka | 2010 | Special Educational Needs (SEN) | Non-government organization | 28 | 100 | 30 | 28 |
| 18 | 22 | Noakhali Buddhi Protibondhi and Autistic School | Chattogram | 2000 | Special Educational Needs (SEN) | Government organization | 21 | 50 | 22 | 21 |
| 19 | 23 | Buddhi Protibondi School | Cumilla | 1984 | Special Educational Needs (SEN) | Government organization | 20 | 55 | 20 | 20 |
| 20 | 24 | Dhrubotara Welfare Society | Dhaka |  | Special Educational Needs (SEN) | Non-government organization | 23 | 67 | 25 | 23 |
| 21 | 25 | Prottasha Center for Autism Care | Dhaka | 2018 | Special Educational Needs (SEN) | Non-government organization | 24 | 80 | 25 | 24 |
| 22 | 31 | Rainbow Autism Care Foundation | Dhaka | 2017 | Special Educational Needs (SEN) | Non-government organization | 5 | 15 | 6 | 5 |
