# Supplementary material for: Socioeconomic and demographic risk factors of autism spectrum disorder among children and adolescents in Bangladesh: Evidence from a cross-sectional study in 2022
Source: PLoS One. 2023 Aug 4;18(8):e0289220. doi: 10.1371/journal.pone.0289220 (PMC10403138; doi:10.1371/journal.pone.0289220)
Supplement: S2 Appendix — (DOCX) [file pone.0289220.s002.docx]

**S2 Appendix:** The literature reviewed for the construction of the questionnaire.

**Table A** Source of literature of neonatal characteristics of children with autism spectrum disorder

| **Variables** | **Categories** |
| --- | --- |
| Gender[1]–[3] | Female |
|  | Male |
| Age[1]–[3] | 2-4 Years |
|  | 5-10 Years |
|  | 11-15 Years |
|  | 16-18 Years |
| Birth weight[1], [2], [4] | < 2.5kg |
|  | ≥ 2.5 kg |
| Birth order[2], [5]–[7] | Firstborns |
|  | Later born |
| Premature birth[1], [8], [9] | No |
|  | Yes |
| Birth asphyxia[4], [9], [10] | No |
|  | Yes |
| Breastfeeding[1], [8], [11] | < 6 months |
|  | 6 to 12 months |
|  | > 12 months |

**Table B** Source of literature of parental scoio-economic and demographic characteristics of autism spectrum disorder

| **Variables** | **Categories** |
| --- | --- |
| Fathers' age at the time of birth[1], [3], [8], [9] | < 40 years |
|  | ≥ 40 years |
| Mothers' age at the time of birth[1], [3], [8], [9] | < 21 years |
|  | 21-35 years |
|  | >35 years |
| Socio economic status[3], [4], [8] | Low |
|  | Middle |
|  | Higher |
| Father's education level[2], [4], [8] | Less than secondary level |
|  | Secondary level |
|  | Higher secondary level |
|  | Graduate |
|  | Higher education |
| Mother's education level[2]–[4], [8] | Less than secondary level |
|  | Secondary level |
|  | Higher secondary level |
|  | Graduate |
|  | Higher education |
| Father's occupation[2], [4] | Not Employed |
|  | Private service |
|  | Government service |
|  | Teacher |
|  | Others |
| Mother's occupation[2], [4] | Housewife |
|  | Private service |
|  | Government service |
|  | Teacher |
|  | Others |
| Family types[8] | Nuclear family |
|  | Joint family |
| Family history of autism disorder[1], [2], [8] | No family history of ASD |
|  | Family history of ASD |
|  | Sibling history of ASD |
| Father illness[8] | No |
|  | Diabetics |
|  | Others |
| Consanguinity[1], [8] | Not related |
|  | First-degree relative |
| Threatened abortion 20 weeks[8], [12] | No |
|  | Yes |
| Specific illness during pregnancy[8], [13]–[15] | No |
|  | Diabetics |
|  | Thyroid |
|  | Others |
| Psychological stress of mother during pregnancy[7], [8], [16] | No |
|  | Yes |
| Maternal history of specific drug use during pregnancy[4], [8] | No |
|  | Yes |
| Poor nutrition during pregnancy[8] | No |
|  | Yes |
| Vitamin D deficit[17]–[20] | No |
|  | Yes |
| Mineral deficiencies[21], [22] | No |
|  | Yes |
| Type of delivery[2], [6], [8] | Vaginal delivery |
|  | Cesarean section |

- Some references successfully used CARS to determine the prevalence and risk factors of ASD [2], [5], [8], [20], [23]–[27].

**Reference**

[1] S. Slama, W. Bahia, I. Soltani, N. Gaddour, and S. Ferchichi, “Risk factors in autism spectrum disorder: A Tunisian case-control study,” *Saudi J. Biol. Sci.*, vol. 29, no. 4, pp. 2749–2755, 2022, doi: 10.1016/j.sjbs.2021.12.059.

[2] A. M. Yousef *et al.*, “Prevalence and risk factors of autism spectrum disorders in preschool children in Sharkia, Egypt: a community-based study,” *Middle East Curr. Psychiatry*, vol. 28, no. 1, 2021, doi: 10.1186/s43045-021-00114-8.

[3] T. K. Bhasin and D. Schendel, “Sociodemographic risk factors for autism in a US metropolitan area,” *J. Autism Dev. Disord.*, vol. 37, no. 4, pp. 667–677, 2007, doi: 10.1007/s10803-006-0194-y.

[4] C. Khaiman, K. Onnuam, S. Photchanakaew, W. Chonchaiya, and K. Suphapeetiporn, “Risk factors for autism spectrum disorder in the Thai population,” *Eur. J. Pediatr.*, vol. 174, no. 10, pp. 1365–1372, 2015, doi: 10.1007/s00431-015-2544-2.

[5] N. Banerjee and P. Adak, “Birth related parameters are important contributors in autism spectrum disorders,” *Sci. Rep.*, vol. 12, no. 1, pp. 1–10, 2022, doi: 10.1038/s41598-022-18628-4.

[6] J. A. Andoy Galvan *et al.*, “Mode of delivery, order of birth, parental age gap and autism spectrum disorder among Malaysian children: A case-control study,” *Heliyon*, vol. 6, no. 10, p. e05068, 2020, doi: 10.1016/j.heliyon.2020.e05068.

[7] G. Russell, C. Steer, and J. Golding, “Social and demographic factors that influence the diagnosis of autistic spectrum disorders,” *Soc. Psychiatry Psychiatr. Epidemiol.*, vol. 46, no. 12, pp. 1283–1293, 2011, doi: 10.1007/s00127-010-0294-z.

[8] B. Geetha, C. Sukumar, E. Dhivyadeepa, J. K. Reddy, and V. Balachandar, “Autism in India: a case–control study to understand the association between socio-economic and environmental risk factors,” *Acta Neurol. Belg.*, vol. 119, no. 3, pp. 393–401, 2019, doi: 10.1007/s13760-018-01057-4.

[9] M. P. Mamidala *et al.*, “Prenatal, perinatal and neonatal risk factors of Autism Spectrum Disorder: A comprehensive epidemiological assessment from India,” *Res. Dev. Disabil.*, vol. 34, no. 9, pp. 3004–3013, 2013, doi: 10.1016/j.ridd.2013.06.019.

[10] E. Hisle-Gorman, A. Susi, T. Stokes, G. Gorman, C. Erdie-Lalena, and C. M. Nylund, “Prenatal, perinatal, and neonatal risk factors of autism spectrum disorder,” *Pediatr. Res.*, vol. 84, no. 2, pp. 190–198, 2018, doi: 10.1038/pr.2018.23.

[11] S. S. Field, “Interaction of genes and nutritional factors in the etiology of autism and attention deficit/hyperactivity disorders: A case control study,” *Med. Hypotheses*, vol. 82, no. 6, pp. 654–661, 2014, doi: 10.1016/j.mehy.2014.02.021.

[12] Y. Yang *et al.*, “Anesthesia, sex and miscarriage history may influence the association between cesarean delivery and autism spectrum disorder,” *BMC Pediatr.*, vol. 21, no. 1, pp. 1–10, 2021, doi: 10.1186/s12887-021-02518-1.

[13] G. Xu, J. Jing, and K. Bowers, “Maternal Diabetes and the Risk of Autism Spectrum Disorders in the Offspring : A Systematic Review and Meta-Analysis,” pp. 766–775, 2014, doi: 10.1007/s10803-013-1928-2.

[14] D. Getahun *et al.*, “Association between maternal hypothyroidism and autism spectrum disorders in children,” *Nat. Publ. Gr.*, vol. 83, no. 3, pp. 580–588, 2018, doi: 10.1038/pr.2017.308.

[15] A. Ornoy, L. Weinstein-Fudim, and Z. Ergaz, “Prenatal factors associated with autism spectrum disorder (ASD),” *Reprod. Toxicol.*, vol. 56, pp. 155–169, Aug. 2015, doi: 10.1016/j.reprotox.2015.05.007.

[16] D. Q. Beversdorf, H. E. Stevens, and K. L. Jones, “Prenatal Stress, Maternal Immune Dysregulation, and Their Association With Autism Spectrum Disorders,” *Curr. Psychiatry Rep.*, vol. 20, no. 9, 2018, doi: 10.1007/s11920-018-0945-4.

[17] M. G. Petruzzelli *et al.*, “Vitamin D Deficiency in Autism Spectrum Disorder: A Cross-Sectional Study,” *Dis. Markers*, vol. 2020, 2020, doi: 10.1155/2020/9292560.

[18] C. Magnusson *et al.*, “Maternal vitamin D deficiency and the risk of autism spectrum disorders: population-based study,” *BJPsych Open*, vol. 2, no. 2, pp. 170–172, 2016, doi: 10.1192/bjpo.bp.116.002675.

[19] J. J. Cannell, “Autism and vitamin D,” *Med. Hypotheses*, vol. 70, no. 4, pp. 750–759, 2008, doi: 10.1016/j.mehy.2007.08.016.

[20] K. Saad *et al.*, “Vitamin D status in autism spectrum disorders and the efficacy of vitamin D supplementation in autistic children,” *Nutr. Neurosci.*, vol. 19, no. 8, pp. 346–351, 2016, doi: 10.1179/1476830515Y.0000000019.

[21] N. L. R. Indika *et al.*, “The Rationale for Vitamin, Mineral, and Cofactor Treatment in the Precision Medical Care of Autism Spectrum Disorder,” *J. Pers. Med.*, vol. 13, no. 2, 2023, doi: 10.3390/jpm13020252.

[22] M. Guo *et al.*, “Vitamin and mineral status of children with autism spectrum disorder in Hainan Province of China: associations with symptoms,” *Nutr. Neurosci.*, vol. 23, no. 10, pp. 803–810, 2020, doi: 10.1080/1028415X.2018.1558762.

[23] C. Chlebowski, J. A. Green, M. L. Barton, and D. Fein, “Using the childhood autism rating scale to diagnose autism spectrum disorders,” *J. Autism Dev. Disord.*, vol. 40, no. 7, pp. 787–799, 2010, doi: 10.1007/s10803-009-0926-x.

[24] A. El-Ansary, G. Bjørklund, A. M. Khemakhem, L. Al-Ayadhi, S. Chirumbolo, and A. Ben Bacha, “Metabolism-Associated Markers and Childhood Autism Rating Scales (CARS) as a Measure of Autism Severity,” *J. Mol. Neurosci.*, vol. 65, no. 3, pp. 265–276, 2018, doi: 10.1007/s12031-018-1091-5.

[25] S. Gunes, O. Ekinci, and T. Celik, “Iron deficiency parameters in autism spectrum disorder: Clinical correlates and associated factors,” *Ital. J. Pediatr.*, vol. 43, no. 1, pp. 1–6, 2017, doi: 10.1186/s13052-017-0407-3.

[26] H. Y. Dong, J. Y. Feng, H. H. Li, X. J. Yue, and F. Y. Jia, “Non-parental caregivers, low maternal education, gastrointestinal problems and high blood lead level: predictors related to the severity of autism spectrum disorder in Northeast China,” *BMC Pediatr.*, vol. 22, no. 1, pp. 1–8, 2022, doi: 10.1186/s12887-021-03086-0.

[27] Q. Zhang *et al.*, “Neurodevelopmental domain characteristics and their association with core symptoms in preschoolers with autism spectrum disorder in China: a nationwide multicenter study,” *BMC Psychiatry*, vol. 22, no. 1, pp. 1–9, 2022, doi: 10.1186/s12888-022-04028-5.
