# Supplementary material for: Socioeconomic and demographic risk factors of autism spectrum disorder among children and adolescents in Bangladesh: Evidence from a cross-sectional study in 2022
Source: PLoS One. 2023 Aug 4;18(8):e0289220. doi: 10.1371/journal.pone.0289220 (PMC10403138; doi:10.1371/journal.pone.0289220)
Supplement: S3 Appendix — (DOCX) [file pone.0289220.s003.docx]

**S3 Appendix:** Assessment of socioeconomic status.

Socioeconomic status was measured by key variables like parents’ education, parents’ occupation, family types (joint/nuclear), present residents, and monthly family income. Monthly family income was expressed in Bangladeshi Taka (BDT) and divided into three categories- 10,000–15,000 BDT, 16,000–40,000 BDT and >40,000 BDT. Finally, socioeconomic status was categorized into three classes- low, middle, and high.

**Socioeconomic index**

Socioeconomic index = (1/2*social index) + (1/2*economic index)

Social Index = (1/2* demographic index) + (1/2* educational index)

**Demographic index**

We considered two factors to construct demographic index

| **Variable** | **Value** |
| --- | --- |
| Family types | Joint= 1  Nuclear= 2 |
| Type of residence | Rural = 1  Sub-urban= 2  Urban = 3 |

Demographic index = Actual value- minimum value/ Maximum value- minimum value

**Educational Index**

Highest education of the respondents was categorized into 8 with values as following

| **Category** | **Value** |
| --- | --- |
| Less than secondary level  Secondary level  Higher secondary level  Graduate  Higher education (post-graduate) | 5  10  12  16  17 |

Individual educational index = Actual value/ Maximum value

Total educational index including husband and wife’s education

Education index = (1/2* husband’s educational index) + (1/2* wife’s educational index).

**Economic Index**

We assumed monthly family income and occupation to calculate the economic index

| **Variable** | **Value** |
| --- | --- |
| Income | 10,000–15,000 BDT = 1  16,000–40,000 BDT = 2  Above 40,000 BDT = 3 |
| Occupation | Unemployed = 1  Housewife= 2  Private/ Government job= 3 |

The formula used to calculate the economic index was.

Economic index = Actual value- minimum value/ Maximum value- minimum value

Using the above formula the range of total SES score lied between 0.15 to 1.5

Low SES= The value for low category was 0.1 to 0.5

Middle SES= participants with score between 0.6 and 1 defined as middle class.

High SES= The range of values for high category was 1.1 to 1.5
