# Supplementary material for: Socioeconomic and demographic risk factors of autism spectrum disorder among children and adolescents in Bangladesh: Evidence from a cross-sectional study in 2022
Source: PLoS One. 2023 Aug 4;18(8):e0289220. doi: 10.1371/journal.pone.0289220 (PMC10403138; doi:10.1371/journal.pone.0289220)
Supplement: S4 Appendix — (DOCX) [file pone.0289220.s004.docx]

**S4 Appendix:** Items modified and added in the CARSc from CARS, and calculation of CARSc.

**Table A** CARS items proposed by Schopler, E. et. al, (1980), and selected items to prepare CARSc for this study along with reliability and validity.

| Childhood Autism Rating Scale (CARS)  Items [[Schopler, E., et. al. (1980)]](https://link.springer.com/article/10.1007/BF02408436) [1[ | Childhood Autism Rating Score (CARSc) | | |
| --- | --- | --- | --- |
|  | Selected Items for this study | ^Item-total Correlation£^ | Reliability |
| 1. Human relatedness^b^ | 1. Imitation^a^ | .421** |  |
| 1. Imitation^a^ | 2. Emotional Response^b^ | .447** |  |
| 1. Affect^e1^ | 3. Visual Response^c^ | .386** | Cronbach's Alpha =  0.68 |
| 1. Use of body^e2^ | 4. Listening Response^d^ | .347** |  |
| 1. Relation to objects^e3^ | 5. Test, smell, and touch response^e^ | .411** |  |
| 1. Adaptation to change^f2^ | 6. Fear and nervous^f^ | .373** |  |
| 1. Visual responsiveness^c^ | 7. Verbal communication^g^ | .321** |  |
| 1. Auditory responsiveness^d^ | 8. Activity level^h^ | .411** |  |
| 1. Near receptor responsiveness^e4^ | 9. Level of intellectual Response^i^ | .346** |  |
| 1. Anxiety reaction^f1^ | 10. General Impression^j^ | .591** |  |
| 1. Verbal communication^g1^ | Scale:- 0: Normal, 1: Mildly Abnormal, 2: Moderately Abnormal, 3: Severely Abnormal | | |
| 1. Nonverbal communication^g2^ |  |  |  |
| 1. Activity level^h^ |  | | |
| 1. Intellectual consistency^i^ |  |  |  |
| 1. Global impression^j^ |  |  |  |
| Scale:- 1: Normal, 2: Mildly Abnormal,  3: Moderately Abnormal,  4. Severely Abnormal | |  |  |
| Note: ** Significant at 5% level, ^£^ validity of the items is measured by correlating the total score with the individual items scale. ^a, b, c, d, h, i, j^ are the items considered in this study from Schopler, E. et. al (1980)[1]. Item ^e1^ was removed, and ^e2, e3, and e4^ were modified to item ^e^ because these items altogether indicate the modified item ^e^ in this study. The items ^f1 and f2^ were modified to ^f^ as ^f1 and f2^ indicate nearly the same meaning. This study considered ^g1^ rather than ^g2^, as ^g2^ is opposite to ^g1,^ and for calculating CARSc score, there should be no opposite items. Hence, item ^g2^ is eliminated. | | | |

**Calculation of Childhood Autism Rating Score (CARSc) for a single observation/unit (a child)**

**Table B** Calculation of CARSc for a single observation

| CARSc Items ($\boldsymbol{i}$**)** | **0** | **1** | **2** | **3** | **Value (**$\boldsymbol{v}_{\boldsymbol{ij}}\boldsymbol{)}$ | **Total Score**  $\boldsymbol{TAS=}\sum_{\boldsymbol{i}}^{\boldsymbol{10}} \sum_{\boldsymbol{j=0}}^{\boldsymbol{3}} \boldsymbol{v}_{\boldsymbol{ij}}$ | **CARSc =**  $\frac{\boldsymbol{TAS}}{\boldsymbol{TAS}_{\boldsymbol{max}}}$ |
| --- | --- | --- | --- | --- | --- | --- | --- |
| 1. Imitation |  |  | **√** |  | **2** ($v_{12})$ | **2+3+0+2+1+**  **2+1+2+2+1 =**  **16** | **16/30 = 0.5333** |
| 2. Emotional Response | $j=3$ | |  | **√** | **3 (**$v_{23})$ |  |  |
| 3. Visual Response | **√** |  |  |  | **0** ($v_{30})$ |  |  |
| 4. Listening Response |  |  | **√** |  | **2** ($v_{42})$ |  |  |
| 5. Test, smell, and touch response |  | **√** |  |  | **1** ($v_{51})$ |  |  |
| 6. Fear and nervous | $j=2$ | | **√** |  | **2** ($v_{62})$ |  |  |
| 7. Verbal communication |  | **√** |  |  | **1** ($v_{71})$ |  |  |
| 8. Activity level |  |  | **√** |  | **2** ($v_{82})$ |  |  |
| 9. Level of intellectual Response |  |  | **√** |  | **2** ($v_{92})$ |  |  |
| 10. General Impression |  | **√** |  |  | **1** ($v_{101})$ |  |  |

**Note: √** is the Response for a child

Table B shows that the CARSc for that particular child is 0.5333.

**Here,** $\boldsymbol{TAS}_{\boldsymbol{max}}$ **=** $\boldsymbol{TAS=}\sum_{\boldsymbol{i}}^{\boldsymbol{10}} \sum_{\boldsymbol{j=3}} \boldsymbol{v}_{\boldsymbol{ij}}$ **= 30, if** $j=3$ (Severely Abnormal) for all $i$.

$\boldsymbol{TAS}_{\boldsymbol{min}}$ **=** $\sum_{\boldsymbol{i}}^{\boldsymbol{10}} \sum_{\boldsymbol{j=0}} \boldsymbol{v}_{\boldsymbol{ij}}$ **= 0,** If $j=0$ (Normal) for all $i$.

**Calculation of the range of CARSc**

${CARSc}_{min}=\frac{TAS}{{TAS}_{max}}=0$, if the total score (TAS) is 0.

${CARSc}_{max}=\frac{TAS}{{TAS}_{max}}=1$, if the total score (TAS) is 30.

Hence, CARSc lies between 0 and 1, i.e., $0\leq CARSc\leq1$

**Reference**

[1] Schopler E, Reichler RJ, DeVellis RF, Daly K. Toward objective classification of childhood autism: Childhood Autism Rating Scale (CARS). J Autism Dev Disord. 1980;10: 91–103. doi:10.1007/BF02408436
